# Understanding Nonlinear Optical Phenomena in N-Pyrimidinyl Stilbazolium Crystals via a Self-Consistent Electrostatic Embedding – DFT Approach

**DOI:** 10.1021/acsomega.4c04215

**Published:** 2024-07-09

**Authors:** Renato Medeiros, Leandro R. Franco, Francisco A.P. Osório, Clodoaldo Valverde, Marcos A. Castro, Tertius L. Fonseca

**Affiliations:** † Instituto de Física, Universidade Federal de Goiás, Goiânia, Goiás 74690-900, Brazil; ‡ Campus de Ciências Exatas e Tecnológicas, Universidade Estadual de Goiás, Anápolis, Goiás 75001-970, Brazil; § Department of Engineering and Physics, Karlstad University, Karlstad 65188, Sweden; ∥ Universidade Paulista, Goiânia, Goiás 74845-090, Brazil

## Abstract

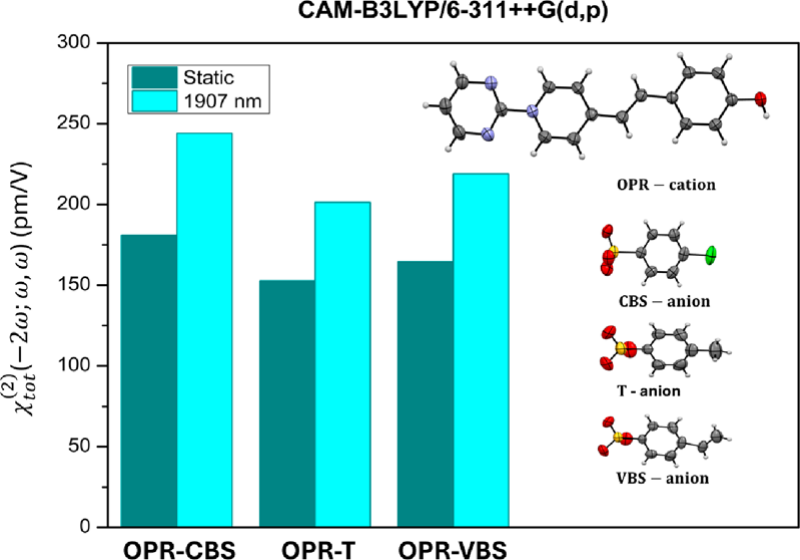

Density functional theory (DFT) and time-dependent density functional theory (TD-DFT) have been used to investigate the nonlinear optical (NLO) properties of phenolic *N*-pyrimidinyl stilbazolium cationic chromophore in its corresponding noncentrosymmetric crystals. Such a cationic chromophore, the OPR (4-(4-hydroxystyryl)-1-(pyrimidin-2-yl)pyridinium), consists of a strong electron donor, the 4-hydroxyphenyl group, and a strong electron acceptor, the *N*-pyrimidinylpyridinium group based on two electron-withdrawing groups. The in-crystal NLO properties were determined by applying a supermolecule approach in combination with an iterative electrostatic scheme, in which the surrounding molecules of a unit cell are represented by point charges. With CAM-B3LYP, our absolute estimates for the largest diagonal component of the second-order nonlinear susceptibility tensor of OPR-based crystals range from 64.00 to 80.34 pm/V in the static regime and from 162.09 to 175.52 pm/V at 1907 nm. These values are significant when compared to those of benchmark stilbazolium-based crystals. Furthermore, the third-order susceptibility, which is related to the nonlinear optical process of the intensity-dependent refractive index, is also significant compared to the results for other organic crystals, such as chalcone derivatives. With TD-CAM-B3LYP, the two-state model effectively explains the similarity in the first hyperpolarizability values in the crystalline phase. This similarity arises from the combination of the oscillator strength and the charge transfer of the crucial transition. Therefore, phenolic organic salt crystals show great promise for various nonlinear optical applications.

## 1 Introduction

There has been considerable interest in the development of highly efficient nonlinear optical organic salt crystals due to their potential applications in high-speed optical modulators, field detectors, frequency conversion, and terahertz wave generation.^1−10^ In particular, salts in which the cation has been designed to have a large molecular hyperpolarizability and where the counterion variation enables the creation of crystals featuring the necessary noncentrosymmetric packing. For instance, one highly polar organic nonlinear optical (NLO) crystals, 4-*N*,*N*-dimethylamino-4′-*N*′-methyl-stilbazolium tosylate (DAST), based on strong Coulomb interactions to achieve noncentrosymmetric crystalline packing, exhibited an second harmonic generation (SHG) efficiency 1000 times greater than of urea, a standard NLO compound.^1^ For this stilbazolium-based organic crystal, the first terahertz-wave generation was observed by using the optical rectification effect induced by 150 fs laser pulses.^3^ Among the DAST derivatives studied using the same cation core structure, we can highlight the 4-*N*,*N*-dimethylamino-4′-*N*′- methyl-stilbazolium 2,4,6-trimethylbenzenesulfonate (DSTMS), and 4-*N*,*N*-dimethylamino-4′-*N*′- phenyl-stilbazolium hexafluorophosphate (DAPSH). Several previous theoretical studies have been conducted to evaluate the NLO properties of these organic salts in crystalline environments.^11−16^ More recently, using a self-consistent electrostatic embedding approach,^17^ we demonstrated that significant interionic interactions in the crystalline state of DAPSH could increase or decrease its molecular hyperpolarizability, β. Thus, the effect of specific interactions can lead to intermolecular charge transfer affecting the β values.

**Figure 1:**
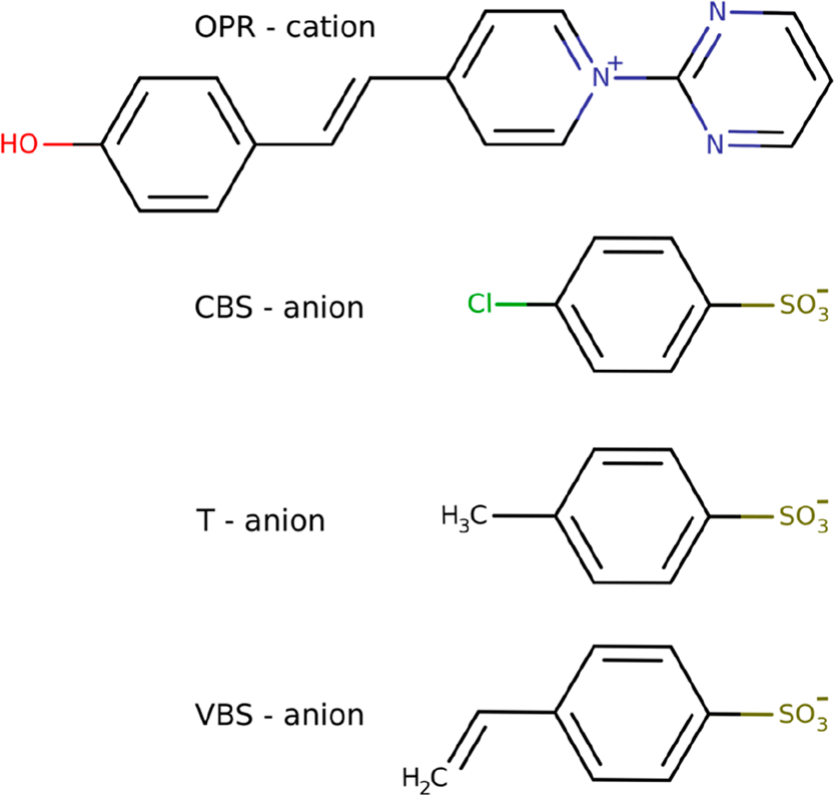
Chemical structures of the OPR-based crystals drawn in their default canonical representations.

**Figure 2:**
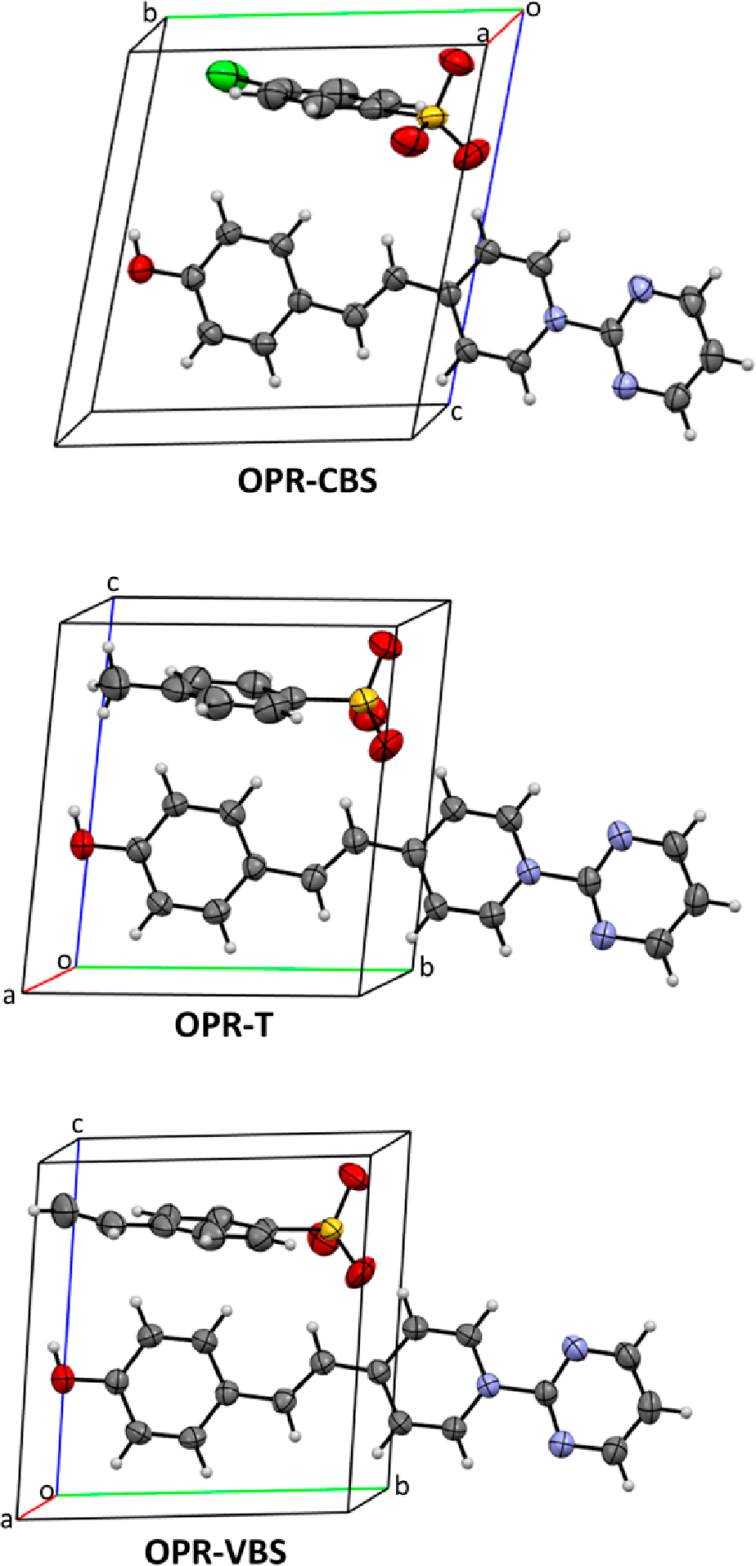
Unit cells of the OPR-based crystals.

Recently, a new class of organic salt crystals having very large macroscopic second-order optical nonlinearity has been prepared for various optical nonlinear applications.^18^ The cationic chromophore, the OPR (4-(4-hydroxystyryl)-1-(pyrimidin-2-yl)pyridinium), was designed to include the N-pyrimidinylpyridinium electron acceptor group, which incorporates two electron-withdrawing groups (EWGs). As counterions for the OPR cationic chromophore, three types of benzenesulfonate anions, 4-vinylbenzenesulfonate (VBS), 4-methylbenzenesulfonate (T), and 4-chlorobenzenesulfonate (CBS) - were employed. The chemical structures of the N-Pyrimidinyl Stilbazolium crystals are presented in Figure 1. All of the OPR-based crystals, with different benzenesulfonate anions (VBS, T, and CBS), exhibit isomorphic crystal structures with space group symmetry *P*1, each containing one pair of molecules (cation and anion) per unit cell as illustrated in Figure 2. Additionally, introducing two EWGs on cationic electron acceptors along with a phenolic electron donor is an effective strategy for achieving a noncentrosymmetric crystal structure with parallel chromophore orientation. Second harmonic generation measurements of OPR crystals are comparable to that of DAST crystals and other various benchmark organic nonlinear optical crystals.^19−21^

The primary aim of this paper is to determine the linear and nonlinear optical properties of highly efficient nonlinear N-pyrimidinyl stilbazolium-salt crystals and to compare these properties with those previously studied for DAPSH, an organic stilbazolium-salt crystal, which exhibits large macroscopic optical nonlinearities. We examine the crystalline effects on the dipole moment, linear polarizability, and first and second hyperpolarizabilities of the OPR-based crystals (OPR-VBS, OPR-T, and OPR-CBS), each with different benzenesulfonate anions, all exhibiting very large macroscopic second-order optical nonlinearity. The computational approach is based on a methodology we developed in previous works,^22−24^ and combines an electrostatic embedding and quantum mechanics (QM) calculations. Estimates of the second- and third-order nonlinear susceptibilities are also presented. Our results for these parameters, when compared with the DASPH crystal, demonstrate that the NLO properties of the OPR-based crystals are remarkably interesting, qualifying these crystals as highly promising materials for various nonlinear optical applications.

## 2 Methodology and Computational Details

To account for the electronic polarization effects arising from the crystalline structure, we utilized an iterative procedure to determine the in-crystal dipole moment (μ) of the OPR-CBS, OPR-T and OPR-VBS molecules within their respective crystalline environments. We start the iterative procedure with partial atomic charges derived from fitting an electrostatic potential on a grid (CHELPG) based on CAM-B3LYP/6-311++G(d,p) charge density of isolated OPR-CBS, OPR-T and OPR-VBS molecules. These CHELPG charges are restrained to reproduce the QM electrostatic potential at various points surrounding the molecules, and they exhibit minimal variation with respect to the basis sets. Since these charges are derived from the QM region, we employ a self-consistent procedure to account for environment effects. Specifically, charges within the QM region are calculated in the presence of the electrostatic embedding of CHELPG charges of the surrounding molecules in the closest unit cells. These QM region charges are then reassigned to the corresponding atomic sites of the cell units, and the charges for the QM region are recalculated in the presence of the newly assigned point charges from the previous iteration of the self-consistent process. Subsequently, these QM charges are reassigned to corresponding atomic sites within the cell units, and the charges within the QM region are recalculated considering the newly assigned point charges from the preceding iteration of the self-consistent process. This iterative cycle continues until the OPR-CBS, OPR-T or OPR-VBS dipole moment converges. Since the pair of molecules in the unit cell is treated explicitly, the main part of the charge transfer is expected to be accounted for. This iterative procedure which consists in describing the inhomogeneous polarizing field of the surrounding molecules has been applied for estimating the linear and nonlinear susceptibilities of a series of nonionic organic crystals^25−27^ In addition, a similar iterative approach has been successfully applied in the study of the polarization of organic molecules in solution.^28−32^

The linear parameters for the OPR-based crystals, as the dipole moment (μ), the average linear polarizability (⟨α⟩), the first-order susceptibility (χ^(1)^), and the linear refractive index were calculated through the expressions
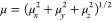
1
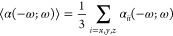
2
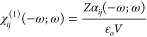
3
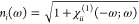
4where *Z* is the number of asymmetric units in the unit cell, *V* the unit cell volume and ε_0_ the vacuum permittivity.

The Cartesian components of the first hyperpolarizability (*β*_*i*_) are given by
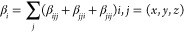
5The magnitude of the total first hyperpolarizability (*β*_tot_) is defined by
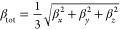
6and the respective second-order susceptibility is given by
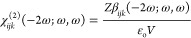
7The average second hyperpolarizability corresponding to the dc-Kerr effect is defined by
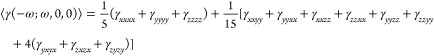
8The average second hyperpolarizability associated with the nonlinear optical process of the intensity dependent refractive index (IDRI) (⟨γ(−ω;ω,ω,−ω)⟩) was calculated by the expression
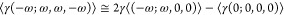
9This is a good approximation for small frequencies^33−35^ compared to the first transition frequency, making the expansion up to second order for γ in power of ω appropriate.

The third-order nonlinear susceptibility (χ^(3)^(−ω;ω,ω,−ω)) of the crystals can be calculated from the equation
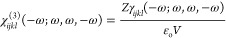
10In all calculations, we used OPR-based crystal structure data without geometry optimization. The OPR-CBS, OPR-T, and OPR-VBS crystals exhibit triclinic symmetry within the *P1* space group. Their respective lattice parameters are as follows: OPR-CBS - *a* = 7.092 Å, *b* = 8.363 Å, *c* = 9.751 Å; OPR-T - *a* = 7.197 Å, *b* = 8.328 Å, *c* = 9.714 Å; OPR-VBS - *a* = 6.919 Å, *b* = 8.561 Å, *c* = 9.733 Å. The unit cell volumes are 527.38 Å^3^ for OPR-CBS and OPR-VBS, and 540.42 Å^3^ for OPR-T. Each unit cell contains one pair of molecules for each crystal type (*Z* = 1). In this study, the crystal field effects on OPR-anion molecules (central QM region) were modeled by including the electrostatic field of the surrounding molecules in the closest unit cells, represented by atomic charges. Therefore, we have considered a cluster of 9 × 9 × 9 unit cells, with each cell containing one pair of OPR-anion molecules. Importantly, we conducted our calculations without imposing periodic boundary conditions. Nonetheless, our trial calculations have demonstrated that a cluster size of at least 7 × 7 × 7 unit cells sufficiently ensures the convergence of the molecular dipole moment within the crystal structure. A previous study on l-arginine phosphate (LAP) monohydrate crystals demonstrated that the dipole moment, linear polarizability, and first hyperpolarizability rapidly converge as the crystal cluster size increases.^22^

All QM calculations were performed with Gaussian 16 program.^36^ Static and dynamic electric properties were calculated by using DFT, while excitation energies were obtained by using TD-DFT at the CAM-B3LYP/6-311++G(d,p) level. The DFT frontier orbitals involved in the dominant transition and change in electron density were obtained with the Gauss view 6.0.16 program.^37^ The experimental structure of the OPR-based crystals determined by X-ray^18^ were used in the calculations.

## 3 Results and Discussion

The basis set effects on the ground state dipole moment [μ, calculated from eq 1] of the OPR-CBS, OPR-T, or OPR-VBS compounds in solid phase are discussed from results illustrated in Table S1 and in Figure S1 (Supporting Information). Comparisons between results obtained using the CAM-B3LYP method with the 6-311++G(d,p) and 6-311G(d,p) basis sets show that the inclusion of diffuse functions has a small impact with variations that do not reach 3% for all compounds, suggesting that reliable results for dipole moment can be obtained with the 6-311G(d,p) basis set. These results are particularly important considering that due to strong Coulomb interactions to achieve noncentrosymmetric crystalline packing, the nature of the diffuse functions can affect the convergence of the dipole moment during the iterative procedure. At the same time, they also suggest that the electrostatic embedding of point charges can be determined with a smaller basis set without the addition of diffuse functions. Consistent with expectation, we notice that the hyperpolarizability values are found to be more sensitive to the basis set effects than the dipole moment values, with variations due to the addition of diffuse functions for *β*_*zzz*_ between 18 and 22%.

Figure S1 (Supporting Information) shows the convergence of the dipole moment for OPR-CBS, OPR-T and OPR-VBS crystals over iterative steps. The in-crystal μ, computed with CAM-B3LYP/6-311++G(d,p), converges to the values of 18.82 D, 17.77 and 17.93 D for OPR-CBS, OPR-T, and OPR-VBS crystals, respectively. These values are 23%, 19% and 21% smaller than the corresponding isolated unit cell values of 24.52 D, 21.87 and 22.70 D. We notice that the effects of crystal polarization for N-Pyrimidinyl Stilbazolium crystals occurs in the opposite direction to that observed for the dipole moment of the asymmetric unit of the DAPSH crystal, estimated to be 49.2 D, 2.5% larger than the isolated value of 48.0 D.^17^ This last converged result is about 100–125% higher than the unit cell results from the OPR crystals. In addition, CAM-B3LYP/6-311++G(d,p) calculations give for the benzenesulfonate anion a CHELPG partial charge of −0.9084e when embedded [and −0.9224 when isolated], while the OPR cation has a CHELPG partial charge of 0.9084e [and 0.9224 when isolated]. This is in line with the ionization state of the benzenesulfonate and the protonation of the 4-(4-hydroxystyryl)-1-(pyrimidin-2-yl)pyridinium in the crystalline structure, indicating some level of charge separation in the ground state.^18^ When comparing with the isolated cation case, we observe that the embedded CHELPG electronic partial charge on the OPR cationic chromophore decreases by 0.0140e. Test calculations and a previous study^38^ indicate that the choice of charge definition has a minor effect, implying a partial charge transfer between ions induced by the electrostatic embedding. For comparison, the embedded CHELPG electronic partial charge on the *N*-phenyl-stilbazolium cationic chromophore in the DAPSH crystal is increased by 0.027e.^17^

Table 1 shows the CAM-B3LYP/6-311++G(d) results for the average linear polarizability [⟨α(−ω;ω)⟩, calculated from eq 2] of isolated and embedded unit cells of the N-Pyrimidinyl Stilbazolium crystals under both, static and dynamic regimes, for the electric field frequencies of ω = 0.0415 au (1097 nm) and ω = 0.0239 au (1907 nm). For all of the OPR-based crystals, the polarization effects of the crystalline environment led to a slight increase in both static and dynamic linear polarizability, which does not reach 5%. This has been observed in previous studies for other crystal systems.^22−24^ It is noteworthy that the influence of the dispersion effect on ⟨α(−ω;ω)⟩ is relatively minor with ⟨α(−ω;ω)⟩/⟨α(0;0)⟩ ratios range from 1.014 to 1.018 for λ = 1907 nm and from 1.048 to 1.061 for λ = and 1097 nm. Also shown in Table 1, the results for the largest component of the linear refractive index [*n*_*y*_ (ω), calculated from eq 4], with the dipole orientation taken as the *z*-direction. At 1907 nm, our CAM-B3LYP results for *n*_*y*_ (ω) of the OPR-CBS, OPR-T and OPR-VBS crystals are equivalent and given by 1.79. These values are comparable to the result for the refractive index of the DAPSH crystal.^17^

**Table 1:** Static and Dynamic Results for the Average Linear Polarizability (in 10^–24^ esu) and the Refractive Index of the OPR-based Crystals Obtained at the CAM-B3LYP/6-311++G(d,p) Level^a^

	OPR-CBS (isolated)	OPR-CBS (embedded)	OPR-T (isolated)	OPR-T (embedded)	OPR-VBS (isolated)	OPR-VBS (embedded)
⟨α(−ω;ω)⟩						
Static	56.48	58.75	55.21	56.23	58.24	58.94
1907 nm	57.34	59.82	56.01	57.12	59.12	59.88
1097 nm	59.31	62.31	57.84	59.17	61.14	62.06
*n*_*y*_ (ω)						
Static	1.66	1.78	1.70	1.78	1.69	1.78
1907 nm	1.67	1.79	1.71	1.79	1.70	1.79
1097 nm	1.69	1.83	1.73	1.83	1.72	1.83

a The dipole orientation is considered in the *z*-direction.

**Figure 3:**
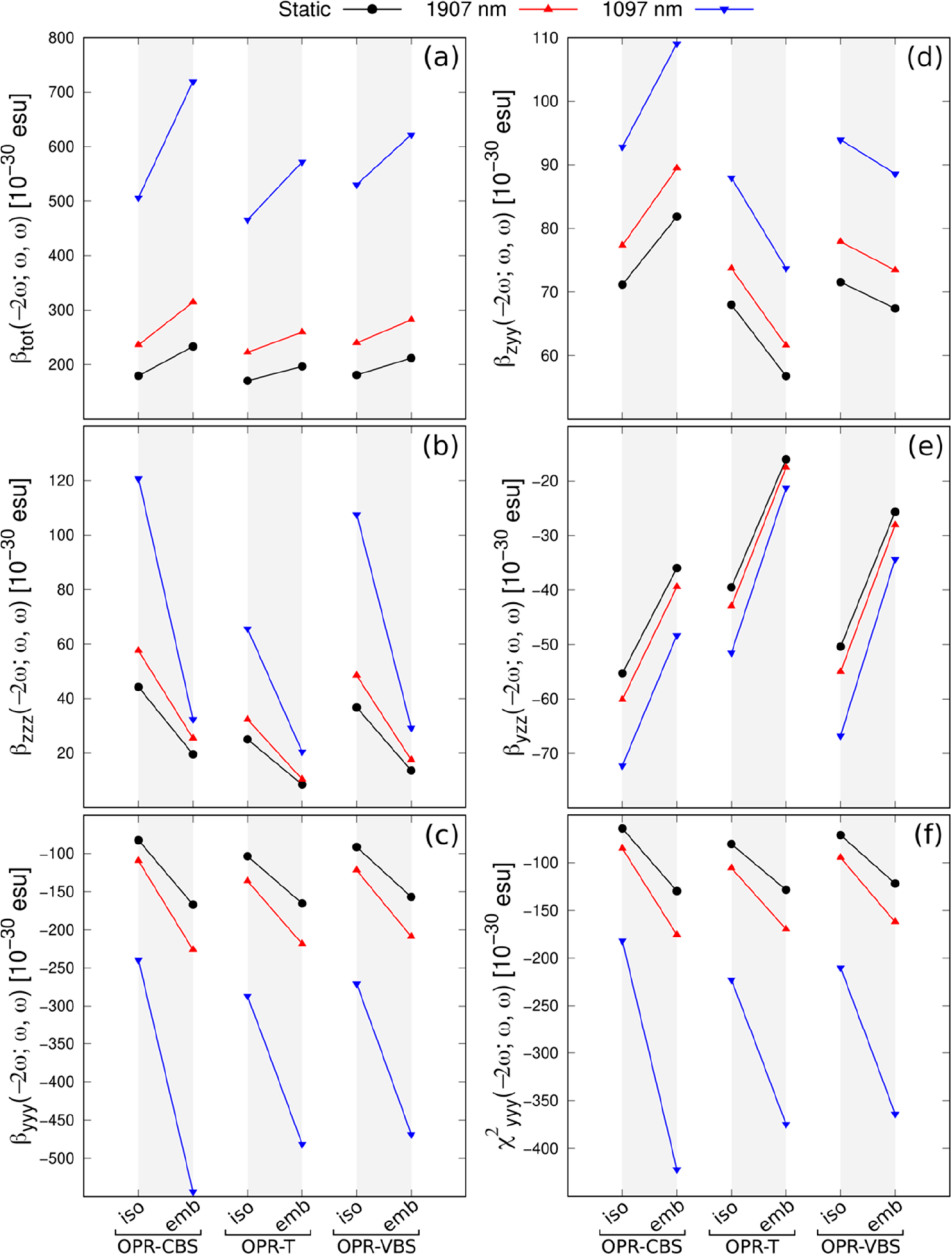
Static and dynamic results for the first hyperpolarizability (in 10^–30^ esu) and the second-order susceptibility (in pm/V) of the OPR-based crystals, obtained at the CAM-B3LYP/6-311++G(d,p) level, with the dipole orientation considered in the *z*-direction: (a) Total first hyperpolarizability; (b) Dipole orientation hyperpolarizability component; (c) largest diagonal hyperpolarizability component; (d, e) off-diagonal hyperpolarizability components; (f) largest diagonal susceptibility component.

An overall look at the results in Table 2 and Figure 3 indicates that the environment polarization effects significantly enhance the total first hyperpolarizability [(*β*_tot_ (−2ω;ω,ω), calculated from eq 6], with a more pronounced effect compared to linear polarizability. For the OPR-based crystals, the embedded static values of *β*_tot_ (0;0,0) exhibit increases between 16 and 30%, relative to their corresponding isolated unit cell values. Slightly increases, with respect to polarization effects, are also observed for the dynamic (at 1907 nm) values of *β*_tot_ (−2ω;ω,ω), especially for the OPR-CBS crystal. We notice that when the dipole orientation is aligned with the *z*-direction, the hyperpolarizability component with the largest value is *β*_*yyy*_ (−2ω;ω,ω), along the main charge-transfer direction of the OPR cationic chromophore. It is found that the off-diagonal hyperpolarizability components (*β*_*yyy*_ (−2ω;ω,ω) and *β*_*yyy*_ (−2ω;ω,ω)) are smaller but non-negligible and may contribute to the macroscopic optical nonlinearity. Consistently, the embedded static and dynamic values of *β*_*yyy*_ (−2ω;ω,ω) exhibit a trend similar to that of *β*_tot_ (−2ω;ω,ω), with significantly larger increases due to crystal polarization, ranging between 60 and 127%. Differently, the impact of the crystalline environment leads to a marked decrease in static and dynamic values of the dipole orientation component, *β*_*zzz*_ (−2ω;ω,ω). At λ = 1907 nm, the dispersion effect for the second harmonic generation is only moderate and independent of the crystalline environment. The *β*_tot_ (−2ω;ω,ω) values for the OPR-CBS, OPR-T, and OPR-VBS crystals are augmented, with respect to static ones, by factors of, respectively, 35%, 32%, and 33%. The dispersion effect has a more meaningful impact on the *β*_tot_ (−2ω;ω,ω) results at 1097 nm, resulting in increases of around 200%, compared to the static results. This may be due to a resonant effect caused by the second harmonic wavelength close to the electronic absorption band. CAM-B3LYP results for the largest diagonal component of the second-order nonlinear susceptibility tensor [χ_*yyy*_^(2)^(−2ω;ω,ω), calculated from eq 7] are also shown in Table 2. Our CAM-B3LYP estimates for the magnitude of χ_*yyy*_^(2)^(−2ω;ω,ω) of OPR-based crystals are between 64.00 and 80.34 pm/V, in the static regime, and between 162.09 and 175.52 pm/V, at 1907 nm. These χ_*yyy*_^(2)^(−2ω;ω,ω) results are very significant, compared to the CAM-B3LYP results reported by DAPSH of 69.40 pm/V (static) and 79.78 pm/V (at 1907 nm).^17^ DAPSH is a material considered very promising for generating THz waves, with excellent NLO properties at 1907 nm, better than of the DAST.^7^

**Table 2:** Static and Dynamic Results for the First Hyperpolarizability (in 10^–30^ esu) and the Second-Order Susceptibility (in ppm/V) of OPR-Based Crystals Obtained at the CAM-B3LYP/6-311++G(d,p) Level^a^

	OPR-CBS (isolated)	OPR-CBS (embedded)	OPR-T (isolated)	OPR-T (embedded)	OPR-VBS (isolated)	OPR-VBS (embedded)
*β*_tot_ (−2ω;ω,ω)						
Static	179.45	233.12	170.19	196.63	180.82	211.96
1907 nm	235.94	314.49	222.48	259.39	239.81	282.04
1097 nm	506.48	719.53	465.64	572.00	530.21	621.85
*β*_*zzz*_ (−2ω;ω,ω)						
Static	44.24	19.56	25.01	8.45	36.74	13.58
1907 nm	57.57	25.39	32.36	10.36	48.46	17.50
1097 nm	120.77	32.44	65.62	20.50	107.60	29.24
*β*_*yyy*_ (−2ω;ω,ω)						
Static	–82.48	–166.97	–103.54	–165.40	–91.44	–156.83
1907 nm	–109.46	–226.18	–135.98	–218.47	–121.86	–208.89
1097 nm	–239.34	–543.51	–286.79	–481.38	–270.41	–468.61
*β*_*zyy*_ (−2ω;ω,ω)						
Static	71.15	81.85	67.99	56.78	71.55	67.41
1907 nm	77.32	89.47	73.71	61.60	77.91	73.42
1097 nm	92.82	109.08	87.97	73.72	93.95	88.61
*β*_*yzz*_ (−2ω;ω,ω)						
Static	–55.28	–35.96	–39.50	–16.03	–50.39	–25.66
1907 nm	–60.12	–39.43	–42.94	–17.51	–55.03	–28.08
1097 nm	–72.25	–48.32	–51.52	–21.28	–66.75	–34.35
χ_*yyy*_^(2)^ (−2ω;ω,ω)						
Static	–64.00	–129.57	–80.34	–128.35	–70.95	–121.70
1907 nm	–84.94	–175.52	–105.52	–169.53	–94.57	–162.09
1097 nm	–181.72	–421.76	–222.55	–374.55	–209.84	–363.64

a The dipole orientation is considered in the *z*-direction.

Our CAM-B3LYP results show that the point charge distribution can affect the first hyperpolarizability of the cationic chromophore through the electrostatic perturbation effect. Regarding only the embedded cationic chromophore of the OPR-CBS, OPR-T, and OPR-VBS crystals, we obtained the *β*_*tot*_(0;0,0) values of 248.9 × 10^–30^ esu, 215.6 × 10^–30^ esu and 2315.6 × 10^–30^ esu which are overestimated by about 10% when compared with the corresponding embedded unit cell results. Consistent with expectation, these results also show a 13–30% increase compared to the hyperpolarizability values of 191–204 × 10^–30^ esu for isolated cationic chromophores reported by Kim et al.,^18^ calculated at the B3LYP/6-311+G(d,p) theory level. These results also demonstrate that this multiscale approach can be used to interpret the impact of the nature and position of the counterion on the linear and nonlinear optical properties of ionic crystals.

**Figure 4:**
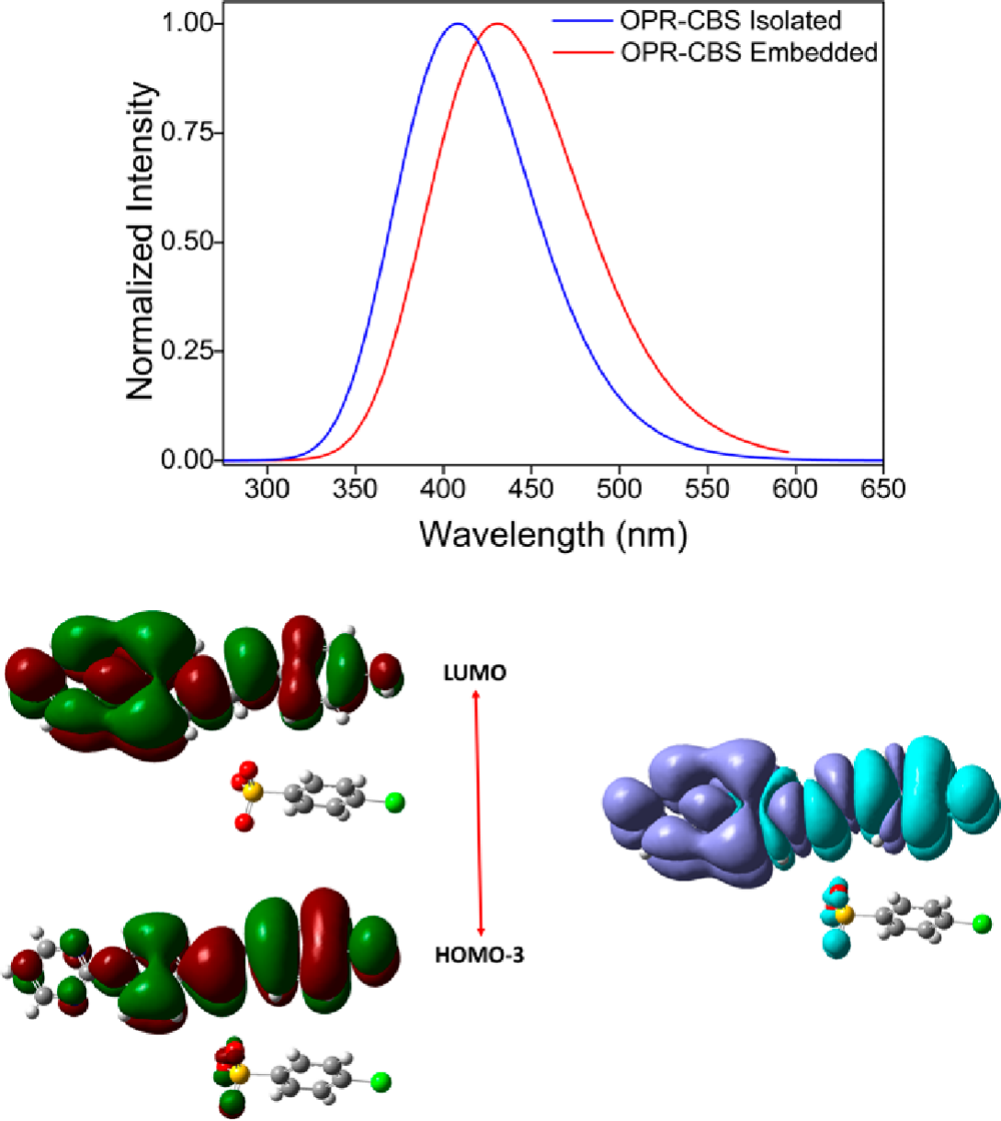
Theoretical UV–Vis absorption spectra of unit cell of the OPR-CBS crystal. Gaussian convolution a with full width at half-maximum (fwhm) of 0.3 eV. Frontier orbitals involved in the dominant transition and change in electron density. The aqua blue (purple) color indicates the region where the electrons are coming (arriving).

The absorption spectra for both the isolated and embedded unit cells of the OPR-CBS crystal show the presence of one dominant transition, indicating that the optical properties are characterized by a π - π* transition (Figure 4). Similar absorption spectra for the OPR-T and the OPR-VBS crystals are shown in Figures S2 and S3 (Supporting Information). TD-CAM-B3LYP/6-311++G(d,p) absorption energy results for this electronic transition are quoted in Table 3. In all of the OPR-CBS, OPR-T, and OPR-VBS crystals, the dominant transition is associated with the excitation from HOMO–3 to LUMO. Analysis of the frontier molecular orbitals for the OPR-CBS crystal reveals that these transitions are marked by intramolecular charge transfer, as depicted in Figure 4. As shown, a major part of HOMO–3 orbital is localized over a strong electron donor, a 4-hydroxyphenyl group. Upon excitation, the LUMO orbital shape corresponds to intramolecular charge transfer from the 4-hydroxyphenyl group to the strong electron acceptor, N-pyrimidinylpyridinium group based on two electron-withdrawing groups. The change in electron density for dominant electronic excitation was calculated as Δρ(*r*) = *ρ*_excited_ (*r⃗*) – *ρ*_ground state_(*r⃗*) and is also illustrated in Figure 4. The pattern highlights the acceptor character of the N-pyrimidinylpyridinium moiety and the donor character of 4-hydroxyphenyl hydroxyphenyl. The patterns of frontier orbitals and Δρ(*r⃗*) are similar for the OPR-T and OPR-VBS crystals (Figures S2 and S3, Supporting Information). The environment polarization effects lead to small red shifts for the π - π* transition of all crystals in going from the isolated phase to the crystalline phase. With CAM-B3LYP, the N-Pyrimidinyl Stilbazolium crystals are colored because they have a maximum absorption peak in the visible range. For the OPR-CBS crystal, the maximum absorbance is predicted to be about 430 nm, so violet/blue is the absorbed color. Thus, the complementary color that is the perceived color is yellow/orange, in line with the experiment.^18^

**Table 3:** CAM-B3LYP Results for the Excitation Energy (*E*_ge_, in eV) and Oscillator Strength (*f*_0_) of the Crucial Electronic Transition, the Ground- and Excited-State Dipole Moment Difference (*μ̅*_*ee*_, in D), and the First Hyperpolarizability of the Two-Level Model (β_tot_^TL^, in 10^–30^ esu) of the OPR-Based Crystals

Crystal	*E*_ge_	*f*_0_	*μ̅*_ee_	β_tot_^TL^
OPR-CBS	2.880	1.225	3.430	56.374
OPR-T	3.007	1.213	3.496	58.047
OPR-VBS	2.970	0.896	4.500	64.992

Having defined the crucial electronic transitions of OPR crystals, we can obtain a qualitative interpretation of the crystalline effects on the first hyperpolarizability by considering the two-level model. We employed the generalized version of Alam et al.^39^ that take into account the effect of dipole alignment and allows us to calculate invariant quantities like *β*_*tot*_, which is given by
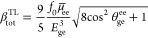
11where *f*_0_ is the oscillator strength, *E*_*ge*_ the transition energy, *μ⃗*_ee_ = |*μ⃗*_ee_ – *μ⃗*_gg_|, and θ_ge_^ee^ is the angle between the vectors *μ⃗*_ge_ and *μ⃗*_ee_, being *μ⃗*_gg_, *μ⃗*_ee_, and *μ⃗*_ge_ the ground state, excited state and transition dipole moment vectors, respectively. This model establishes a link between the first hyperpolarizability and the so-called crucial electronic transitions. Table 3 also reports the spectroscopic factors of the two-level model calculated at the CAM-B3LYP level from crystal geometries obtained from a crystallographic information file without any additional changes to the geometry used in subsequent quantum mechanics calculations. Results for vector components of *μ⃗*_gg_, *μ⃗*_ee_, and *μ⃗*_ge_ are quoted in Table S2, Supporting Information. We clearly notice that the values of β_tot_^TL^ for OPR crystals are similar, in agreement with the *β*_tot_ (0;0,0) values in Table 2, which results from the combination of the oscillator strength and the charge transfer of the crucial transition. Therefore, these findings indicate that *f*_0_ and *μ̅*_ee_ play crucial roles in determining the hyperpolarizability values of the OPR crystals. Notably, a qualitative interpretation of crystalline dependence of *β*_tot_ (0;0,0) within the framework of the two-state model is validated because OPR crystals have a crucial transition with charge-transfer character.^40^

In Table 4, we report the static and dynamic results for the average second hyperpolarizability [⟨γ (−ω;ω,ω,−ω)⟩, calculated from eq 9] and the largest diagonal component of the second hyperpolarizability [*γ*_*yyyy*_ (−ω;ω,ω, −ω)] (related to the third-order NLO processes of IDRI) for the isolated and embedded unit cell of the N-Pyrimidinyl Stilbazolium crystals. As seen with the first hyperpolarizability, the static and dynamic (at 1907 nm) values of ⟨γ (−ω;ω,ω,−ω)⟩ have equivalent increases ranging from 8 to 21% due to crystal packing when compared to the results for isolated unit cells. The most significant increases are observed for the hyperpolarizability of the OPR-CBS crystal. This contrasts with the small impact of the polarization effect on the ⟨γ (−ω;ω,ω,−ω)⟩ values of the asymmetric unit of the DAPSH crystal.^17^ At 1907 nm, the influence of dispersion on ⟨γ (−ω;ω,ω,−ω)⟩ is moderate and independent of the crystalline medium. With CAM-B3LYP, the ⟨γ (−ω;ω,ω,−ω)⟩ values for the OPR-CBS, OPR-T and OPR-VBS crystals are increased by 28%, 25%, and 26%, respectively, compared to their static counterparts. For comparison, our predictions for ⟨γ (-ω;ω,ω,−ω)⟩, at 1907 nm, are between 53 and 80% larger than the ionic organic crystal (VSNS) value, which is estimated to be 258 × 10^–36^ esu. We note that for OPR-crystals our CAM-B3LYP estimates for the largest diagonal component of the third-order nonlinear susceptibility tensor [(χ_*yyyy*_^(3)^ (−ω;ω,ω,−ω), calculated from eq 10] are between 2.64 and 3.01 (10^–20^ m^2^/V^2^), in the static regime, and between 3.42 and 3.80 (10^–20^ m^2^/V^2^), at 1907 nm. For comparison, the results for the average susceptibility, (⟨χ^(3)^(−ω;ω,ω,−ω)⟩), range from 0.66 and 0.77 (10^–20^ m^2^/V^2^) in the static regime and from 0.83 and 0.97 (10^–20^ m^2^/V^2^) at 1907 nm. These values are practically of the same order of magnitude as those reported for DAPSH, which are 0.96 and 1.19 (10^–20^ m^2^/V^2^).^17^

**Table 4:** Static and Dynamic Results for the Second Hyperpolarizability (in 10^–36^ esu) and the third-order susceptibility (in 10^–20^ m^2^/V^2^) of the OPR-Based Crystals Obtained at the CAM-B3LYP/6-311++G(d,p) Level^a^

	OPR-CBS (isolated)	OPR-CBS (embedded)	OPR-T (isolated)	OPR-T (embedded)	OPR-VBS (isolated)	OPR-VBS (embedded)
⟨γ (−ω;ω,ω, −ω)⟩						
Static	294.42	343.14	293.75	316.57	332.05	367.26
1907 nm	364.39	439.97	361.80	395.86	411.77	464.23
1097 nm	553.54	713.85	544.93	612.05	628.12	738.37
*γ*_*yyyy*_ (−ω;ω,ω, −ω)						
Static	484.89	1020.36	693.70	1163.02	614.71	1141.67
1907 nm	607.85	1322.40	863.67	1468.62	770.29	1450.03
1097 nm	940.64	2174.48	1318.72	2301.56	1191.60	2300.23
χ_*yyyy*_^(3)^ (−ω;ω,ω, −ω)						
Static	1.26	2.64	1.80	3.01	1.59	2.96
1907 nm	1.57	3.42	2.24	3.80	1.99	3.75
1097 nm	2.43	5.63	3.41	5.96	3.08	5.95

a The dipole orientation is considered in the *z*-direction.

## 4 Conclusion

We employed an iterative electrostatic polarization scheme to determine the dipole moment of the unit cell of the OPR-based crystals. Our investigation offers a first estimation of the macroscopic susceptibilities, considering the substantial influence of electrostatic interactions on the crystal arrangement. Specifically, we used results from the polarized unit cell to estimate the macroscopic susceptibilities. Our results reveal that the dipole moment of the unit cell for the OPR-CBS, OPR-T, and OPR-VBC crystals, obtained with CAM-B3LYP/6-311++G(d,p), converges to 18.82, 17.77, and 17.93 D, respectively. These values are 23%, 19%, and 21% smaller than the corresponding isolated unit cell values of 24.52 21.87, and 22.70 D. We note that polarization effects have a moderate impact on static and dynamic hyperpolarizabilities, being independent of the crystal environment. Our absolute results for χ_*yyyy*_^(2)^ (−2ω;ω,ω,−ω) of OPR-based crystals are between 64.00 and 80.34 pm/V, in the static regime, and between 162.09 and 175.52 pm/V, at 1907 nm, which are very significant, compared to the CAM-B3LYP results reported by DAPSH of 69.40 pm/V (static) and 79.78 pm/V (at 1907 nm),^17^ a reference stilbazolium-based crystal. Therefore, our findings suggest that OPR-based crystals are highly promising materials for generating THz waves with excellent NLO properties at 1907 nm. In addition, the third-order susceptibility, related to the NLO process of the intensity dependent refractive index, is significant compared with the results for other organic crystals, such as chalcone-derivatives. Our results for χ_*yyyy*_^(3)^ (−ω;ω,ω,−ω) of the OPR-crystals range from 2.64 and 3.01 (10^–20^ m^2^/V^2^) in the static regime and from 3.42 and 3.80 (10^–20^ m^2^/V^2^) at 1907 nm.

## Supporting Information Available

Comparison between the CAM-B3LYP results for dipole moment, linear polarizability, first and second hyperpolarizabilities computed with the 6-311++G(d,p) and 6-311G(d,p) basis sets, and convergence of the total dipole moment of the unit cell of the OPR-CBS, OPR-T ,and OPR-VBS crystals as a function of the iterative step (PDF)
