# Supplementary material for: Understanding Nonlinear Optical Phenomena in N-Pyrimidinyl Stilbazolium Crystals via a Self-Consistent Electrostatic Embedding – DFT Approach
Source: ACS Omega. 2024 Jul 9;9(29):32080–8. doi: 10.1021/acsomega.4c04215 (PMC11270711; doi:10.1021/acsomega.4c04215)
Supplement: Supplementary file 1 — ao4c04215_si_001.pdf [file ao4c04215_si_001.pdf]

Supporting Information

Understanding Nonlinear Optical Phenomena in N-Pyrimidinyl Stilbazolium Crystals via a Self-Consistent Electrostatic Embedding – DFT Approach

Renato Medeiros[a,b], Leandro R. Franco[c], Francisco A.P. Osório[a], Clodoaldo Valverde[b,d], Marcos A. Castro[a] and Tertius L. Fonseca[a,*]

[a]Instituto de Física, Universidade Federal de Goiás, Goiânia-GO, 74690-900, Brazil.

[b]Campus de Ciências Exatas e Tecnológicas, Universidade Estadual de Goiás, Anápolis-GO, 75001-970, Brazil

[c]Department of Engineering and Physics, Karlstad University, 65188 Karlstad, Sweden.

[d]Universidade Paulista, Goiânia-GO, 74845-090, Brazil.

Table S1. CAM-B3LYP results for the static linear and nonlinear optical properties of the OPR-based crystals obtained with the 6-311++G(d,p) and 6-311G(d,p) basis set.

Basis set	OPR-CBS (isolated)	OPR-CBS (embedded)	OPR-T (isolated)	OPR-T (embedded)	OPR-VBS (isolated)	OPR-VBS (embedded)
$\langle\alpha\rangle$ (in 10^{-24}esu)						
6-311++G(d,p)	56.48	58.75	55.21	56.23	58.24	58.94
6-311G(d,p)	52.73	54.76	51.74	52.56	54.45	54.93
β_{tot} (in 10^{-30}esu)						
6-311++G(d,p)	179.45	233.12	170.19	196.63	180.82	211.96
6-311G(d,p)	175.18	223.71	166.31	190.25	177.45	205.57
β_{zzz} (in 10^{-30}esu)						
6-311++G(d,p)	44.24	19.56	25.01	8.45	36.74	13.58
6-311G(d,p)	41.63	16.48	23.55	6.94	34.49	11.53
β_{yyy} (in 10^{-30}esu)						
6-311++G(d,p)	-82.48	-166.97	-103.54	-165.40	-91.43	-156.83
6-311G(d,p)	-82.75	-163.46	-103.48	-162.25	-92.82	-154.77
$\langle\gamma_{\parallel}\rangle$ (in 10^{-36}esu)						
6-311++G(d,p)	294.42	343.14	293.75	316.57	332.05	367.26
6-311G(d,p)	261.23	291.54	263.06	275.92	295.18	322.18

Table S2. CAM-B3LYP results for vector components of ground-state dipole moment ($\vec{\mu}_{gg}$), excited-state dipole moment ($\vec{\mu}_{ee}$) and transition dipole moment ($\vec{\mu}_{ge}$) of the OPR crystals. All values are given in D.

Crystal	$\mu_{gg,x}$	$\mu_{gg,y}$	$\mu_{gg,z}$	$\mu_{ee,x}$	$\mu_{ee,y}$	$\mu_{ee,z}$	$\mu_{ge,x}$	$\mu_{ge,y}$	$\mu_{ge,z}$
OPR-CBS	11.531	-2.812	14.610	8.418	5.200	13.155	-3.349	10.016	-0.819
OPR-T	11.531	-2.812	14.610	8.418	5.200	13.155	-2.637	9.938	-0.807
OPR-VBS	10.918	-1.515	14.142	4.554	6.408	8.893	-3.188	8.312	-0.564

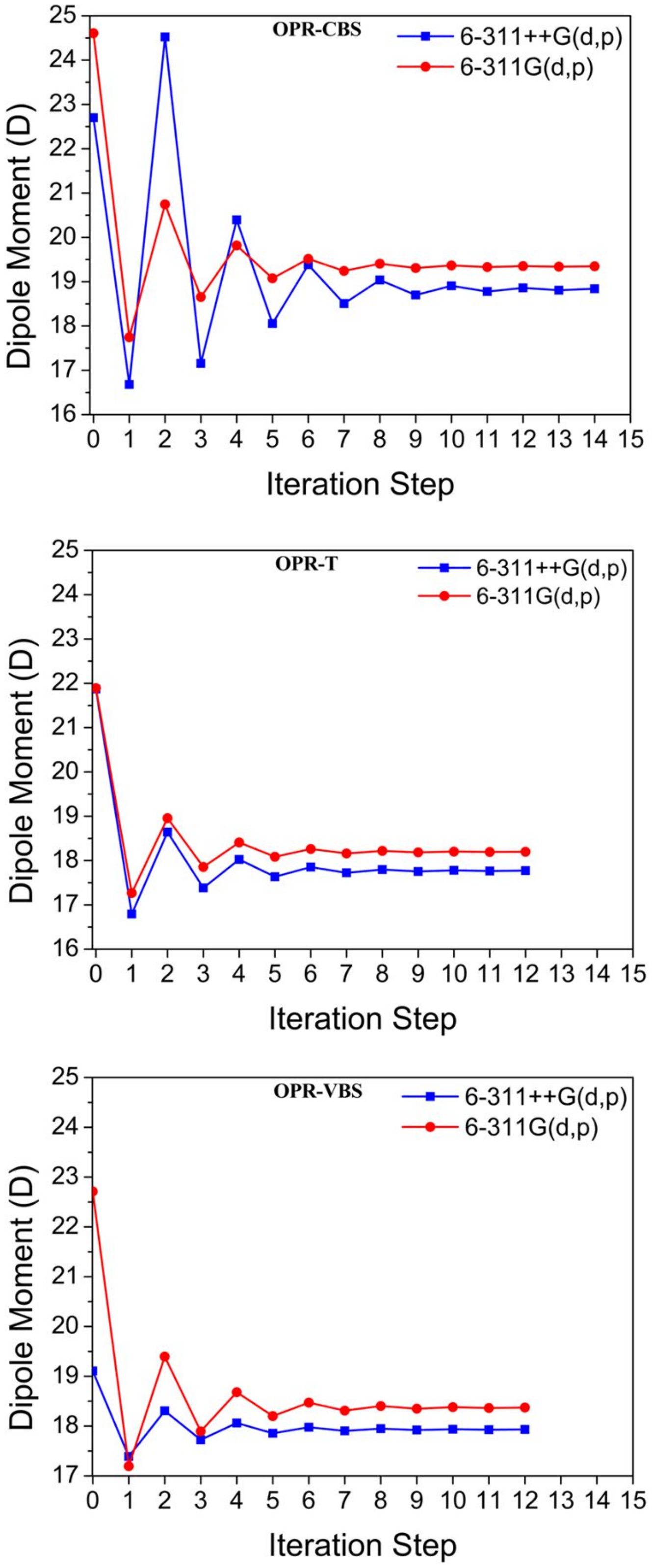

Figure S1. Convergence of dipole moment of the OPR-based crystals as function of the iterative procedure.

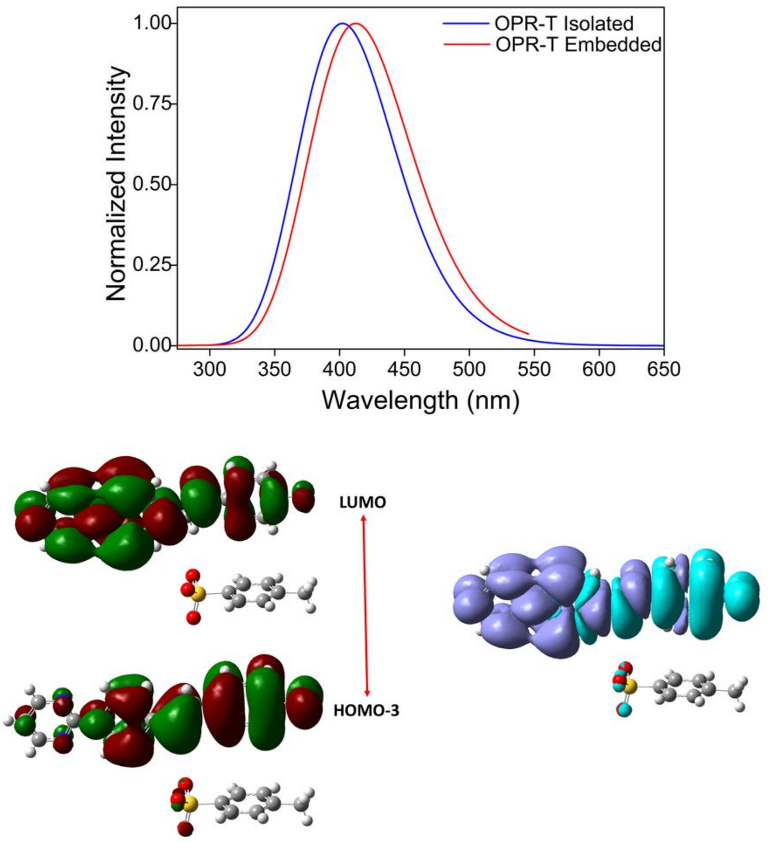

Figure S2. Theoretical UV–Vis absorption spectra of unit cell of OPR-T crystal. Gaussian convolution a with full width at half maximum (FWHM) of 0.3 eV. Frontier orbitals involved in the dominant transition and change in electron density. The aqua blue (purple) color indicates the region where the electrons are coming (arriving).

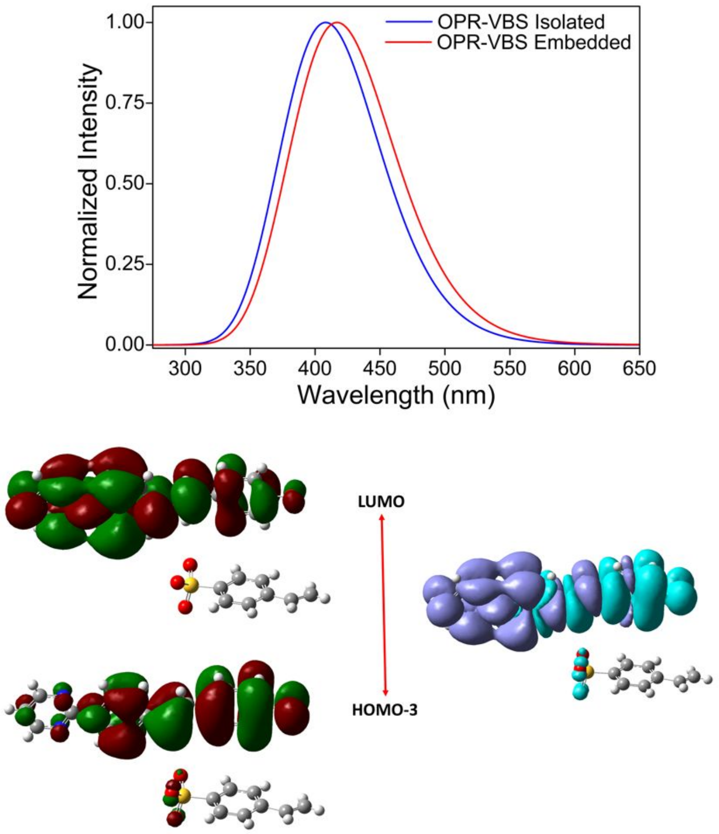

Figure S3. Theoretical UV–Vis absorption spectra of unit cell of OPR-VBS crystal. Gaussian convolution a with full width at half maximum (FWHM) of 0.3 eV. Frontier orbitals involved in the dominant transition and change in electron density. The aqua blue (purple) color indicates the region where the electrons are coming (arriving).
